# Supplementary material for: Structure of the native pyruvate dehydrogenase complex reveals the mechanism of substrate insertion
Source: Nat Commun. 2021 Sep 6;12:5277. doi: 10.1038/s41467-021-25570-y (PMC8421416; doi:10.1038/s41467-021-25570-y)
Supplement: Supplementary file 1 — Supplementary Information [file 41467_2021_25570_MOESM1_ESM.pdf]

Structure of the native pyruvate dehydrogenase complex reveals the mechanism of substrate insertion

Jana Škerlová[1], Jens Berndtsson[1], Hendrik Nolte[2], Martin Ott[1,3], Pål Stenmark[1,4]

[1]Department of Biochemistry and Biophysics, Stockholm University, SE-10691 Stockholm, Sweden
[2]Max-Planck-Institute for Biology of Ageing, Joseph-Stelzmann Str. 9b, 50931 Cologne, Germany
[3]Department of Medical Biochemistry and Cell Biology, University of Gothenburg, SE-40530 Gothenburg, Sweden
[4]Department of Experimental Medical Science, Lund University, SE-22100 Lund, Sweden

Supplementary Information

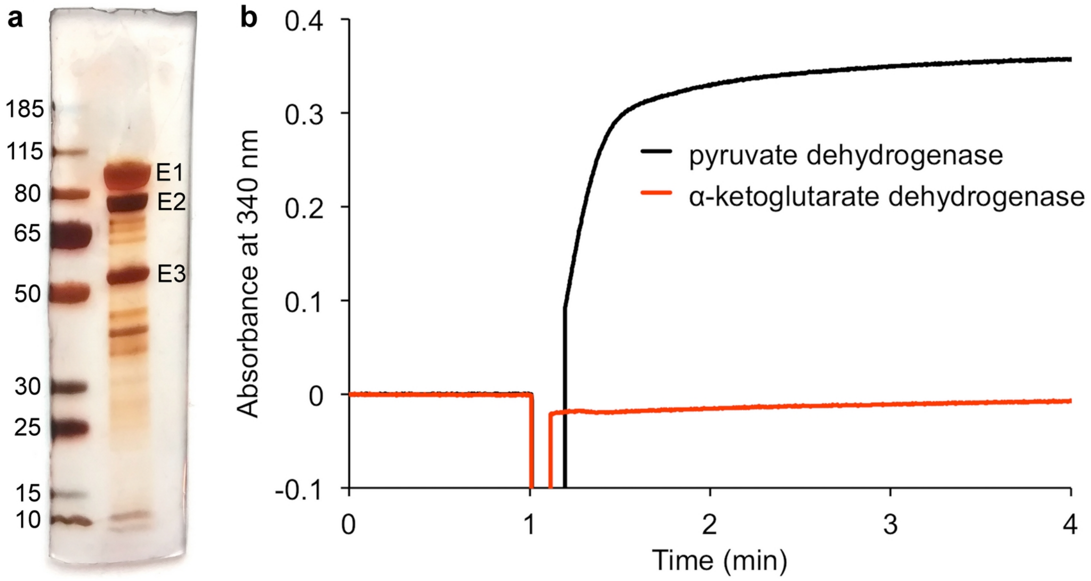

Supplementary Fig. 1 Purity and enzymatic activity of the isolated pyruvate dehydrogenase complex. a Silver-stained SDS-PAGE analysis of the sample used for cryo-EM experiments. (Silver-stained SDS-PAGE analysis was performed twice from two independent purifications). Standard molecular weights in kDa are indicated. **b** Photometric assay of pyruvate dehydrogenase (black) and α-ketoglutarate (red) activities in the isolated pyruvate dehydrogenase complex. The enzyme complex was added to the reaction mixture 60 seconds after the start of the absorbance recording.

Supplementary Table 1 Mass spectrometry analysis.

Top 20 proteins identified in the purified pyruvate dehydrogenase complex sample are listed. Components of the PDHc are highlighted in bold and yellow. Components of the stable ubiquinone-synthesizing metabolon are highlighted in grey. OS – organism, GN – gene name, PSMs – peptide spectrum matches. The complete mass spectrometry analysis data can be found in the Source Data file. The Excel file contains multiple sheets and is accompanied with a description of each column header.

Uniprot ID	Description	Score	Coverage	Unique Peptides	PSMs
**P0AFG8**	**Pyruvate dehydrogenase E1 component OS=*Escherichia coli* (strain K12) GN=*aceE***	**2,942.52**	**83.43**	**88**	**1478**
**P06959**	**Dihydrolipoyllysine-residue acetyltransferase component of pyruvate dehydrogenase complex OS=*Escherichia coli* (strain K12) GN=*aceF***	**2,502.47**	**89.21**	**67**	**1090**
**P0A9P0**	**Dihydrolipoyl dehydrogenase OS=*Escherichia coli* (strain K12) GN=*lpdA***	**1,424.99**	**66.24**	**35**	**631**
P0A6Y8	Chaperone protein DnaK OS=*Escherichia coli* (strain K12) GN=*dnaK*	231.34	67.24	40	83
P0ABB4	ATP synthase subunit beta OS=*Escherichia coli* (strain K12) GN=*atpD*	208.38	66.96	25	70
P0ABB0	ATP synthase subunit alpha OS=*Escherichia coli* (strain K12) GN=*atpA*	203.85	61.21	25	71
P0A6F5	60 kDa chaperonin OS=*Escherichia coli* (strain K12) GN=*groL*	187.92	48.91	22	70
P0A9Q7	Aldehyde-alcohol dehydrogenase OS=*Escherichia coli* (strain K12) GN=*adhE*	152.09	44.67	32	54
P0A9G6	Isocitrate lyase OS=*Escherichia coli* (strain K12) GN=*aceA*	142.56	64.52	20	47
P0AC41	Succinate dehydrogenase flavoprotein subunit OS=*Escherichia coli* (strain K12) GN=*sdhA*	141.53	55.61	23	50
P0A9A6	Cell division protein FtsZ OS=*Escherichia coli* (strain K12) GN=*ftsZ*	125.35	65.27	18	46
P33602	NADH-quinone oxidoreductase subunit G OS=*Escherichia coli* (strain K12) GN=*nuoG*	121.86	33.04	22	41
P0A887	Ubiquinone/menaquinone biosynthesis C-methyltransferase UbiE OS=*Escherichia coli* (strain K12) GN=*ubiE*	115.26	55.38	13	38
P21513	Ribonuclease E OS=*Escherichia coli* (strain K12) GN=*rne*	108.66	34.02	27	42
P0A6E4	Argininosuccinate synthase OS=*Escherichia coli* (strain K12) GN=*argG*	107.85	51.01	20	37
P02942	Methyl-accepting chemotaxis protein I OS=*Escherichia coli* (strain K12) GN=*tsr*	104.76	44.10	18	36
P77488	1-deoxy-D-xylulose-5-phosphate synthase OS=*Escherichia coli* (strain K12) GN=*dxs*	103.85	40.97	21	41
P0AFG3	2-oxoglutarate dehydrogenase E1 component OS=*Escherichia coli* (strain K12) GN=*sucA*	103.71	35.91	25	44
P28635	D-methionine-binding lipoprotein MetQ OS=*Escherichia coli* (strain K12) GN=*metQ*	103.53	58.30	15	32
P0A9S5	Glycerol dehydrogenase OS=*Escherichia coli* (strain K12) GN=*gldA*	100.22	45.23	10	34
:	:	:	:	:	:
P75728	2-octaprenyl-3-methyl-6-methoxy-1,4-benzoquinol hydroxylase OS=*Escherichia coli* (strain K12) GN=*ubiF*	57.23	32.23	8	19
:	:	:	:	:	:
P17993	Ubiquinone biosynthesis O-methyltransferase OS=*Escherichia coli* (strain K12) GN=*ubiG*	44.96	63.75	9	14
:	:	:	:	:	:
P25535	2-octaprenylphenol hydroxylase OS=*Escherichia coli* (strain K12) GN=*ubiI*	39.10	43.75	13	17
:	:	:	:	:	:
Q46868	Uncharacterized protein YqiC OS=*Escherichia coli* (strain K12) GN=*yqiC*	38.87	48.96	8	20
:	:	:	:	:	:
P25534	2-octaprenyl-6-methoxyphenol hydroxylase OS=*Escherichia coli* (strain K12) GN=*ubiH*	31.58	41.07	11	13
:	:	:	:	:	:
P0ADP7	Ubiquinone biosynthesis protein UbiJ OS=*Escherichia coli* (strain K12) GN=*ubiJ*	17.13	19.40	3	7

a

Strain	Sample	Specific activity (mM/min/mg)	Purity factor
K12 (WT)	Lysate	0.8	1.0
	Not bound	0.8	N/A
	Elute	10.7	13.5
K12 (UbiF-FLAG)	Lysate	0.7	1.0
	Not bound	0.7	N/A
	Elute	9.6	14.0

b

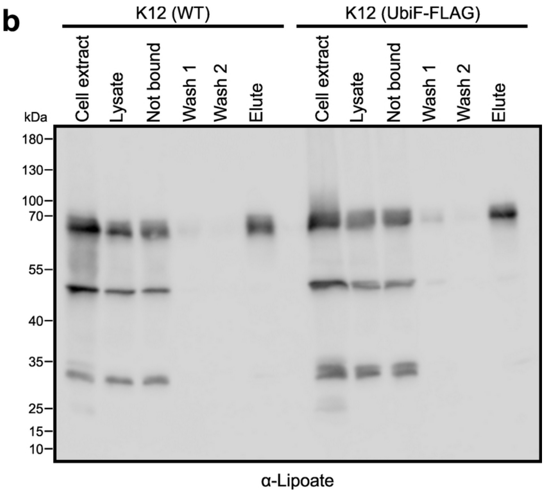

Supplementary Fig. 2 PDHc purification. a Table of specific activity for the lysate, not bound fraction, and elution of the anti-FLAG affinity chromatography purification of the wild-type and UbiF-FLAG *E. coli* K12 strains. The specific activity is the average of two measurements. Purity factor indicates how many times more pure the elution is compared to the lysate. **b** Western blot analysis of anti-FLAG affinity chromatography purification for the wild-type and UbiF-FLAG *E. coli* K12 strains, using an anti-lipoate antibody. The Western blot analysis was performed once.

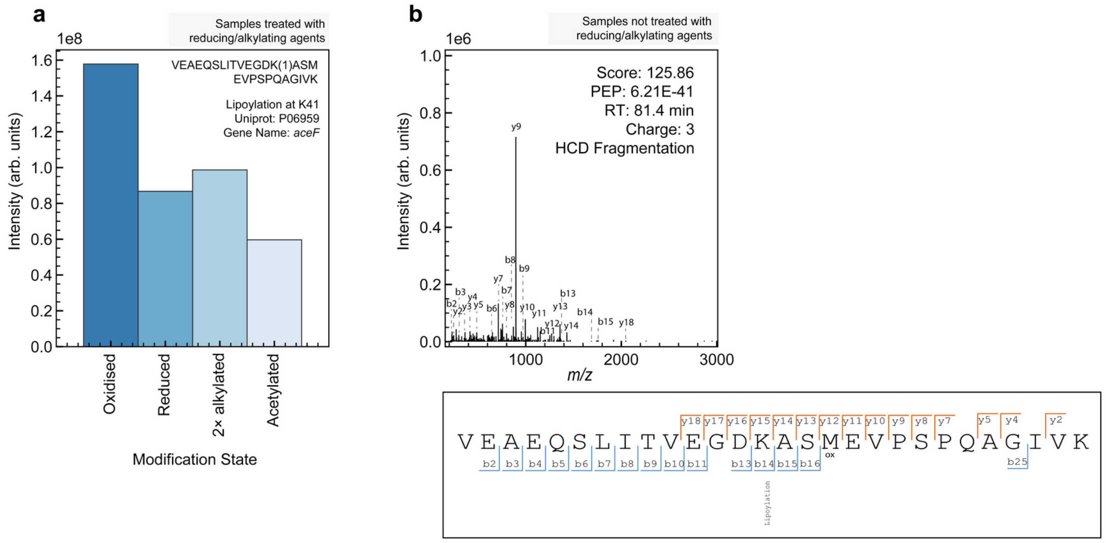

Supplementary Fig. 3 Mass-spectrometry analysis of protein lipoylation. a Intensity of the lipoylation modification at residue K41 of dihydrolipoyl transacetylase in different states (*aceF*, Uniprot: P06959). The potential acetylated/1× alkylated form was not detected. **b** MS/MS spectra for the identification of K41 in samples that were not treated with reducing and alkylating agents prior digestion. Several b- and y- ions are indicated in the spectra. Fragment ions after water loss are not indicated in the spectra. HCD – higher-energy C-trap dissociation, PEP – posterior error probability, RT – retention time.

Supplementary Table 2 Cryo-EM data collection, refinement and validation statistics.

	E. coli E2p (EMD-12104) (PDB 7b9k)		
**Data collection and processing**			
Dataset	1	2	3
Magnification	130,000×	130,000×	130,000×
Voltage (kV)	300	300	300
Electron exposure (e^-/Å^2)	30.00	28.11	28.11
Defocus range (μm)	-0.7 – -1.9	-0.5 – -2.0	-1.1 – -2.9
Pixel size (Å)	1.04	1.04	1.04
Symmetry imposed		O	
Initial particle images (no.)		799,790	
Final particle images (no.)		29,434	
Map resolution (Å)		3.16	
FSC threshold		0.143	
Map resolution range (Å)		2.8 – 7.5 Å	
**Refinement**			
Initial model used (PDB code)		4n72, 1qjo	
Model resolution (Å)		2.8/3.2	
FSC threshold		0.143/0.5	
Map sharpening *B* factor (Å^2)		-102.9	
Model composition			
Non-hydrogen atoms		59,136	
Protein residues		7,680	
B factors (Å^2)			
Protein		91.01	
R.m.s. deviations			
Bond lengths (Å)		0.006	
Bond angles (°)		1.054	
Validation			
MolProbity score		2.01	
Clashscore		13.41	
Poor rotamers (%)		1.11	
Ramachandran plot			
Favored (%)		95.05	
Allowed (%)		4.95	
Disallowed (%)		0	

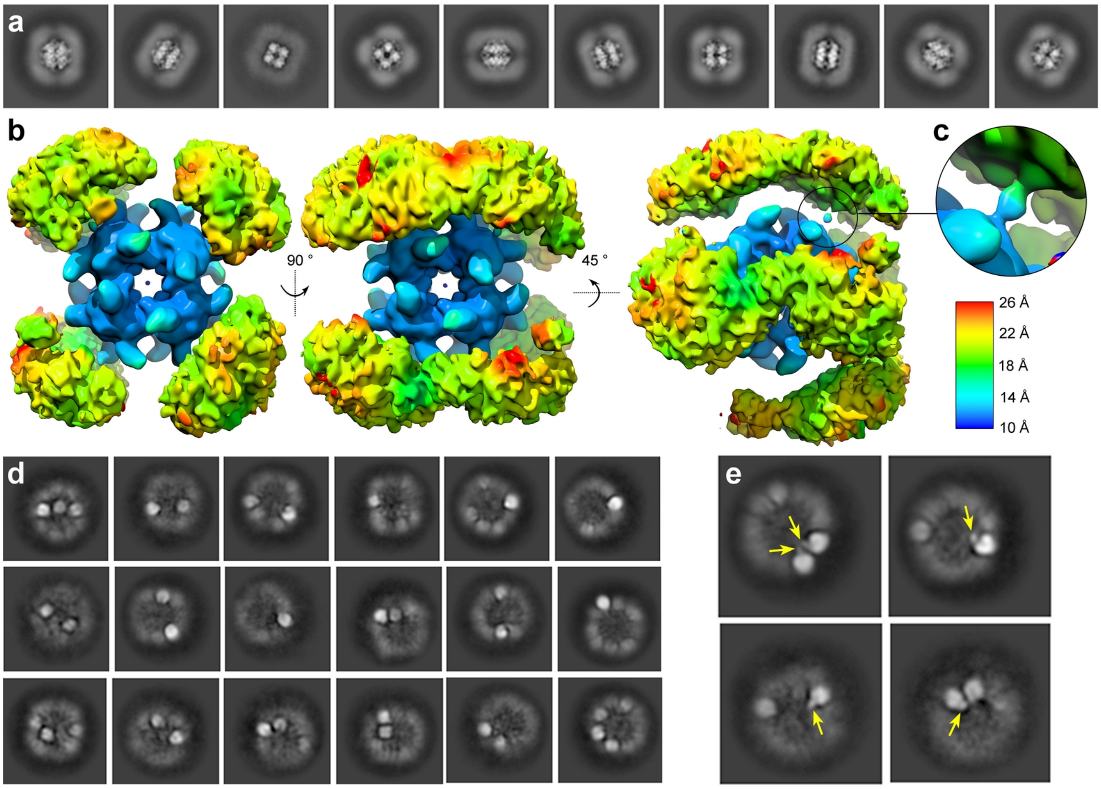

Supplementary Fig. 4 Cryo-EM reconstruction of the *E. coli* pyruvate dehydrogenase complex. **a** 2D classes selected for the 3D reconstruction. **b** cryo-EM map for the whole PDHc colored according to local resolution estimate (FSC = 0.5). **c** cryo-EM map for the linker segment between the catalytic core and the outer shell of the complex (lower map threshold than in panel b). **d** Representative 2D classes from a 2D classification of the particles after the subtraction of the signal for the inner E2p core, which demonstrate the presence of E1p and/or E3 in the outer shell of the complex. **e** Selected 2D classes from the 2D classification described in panel d, which include visible linker segments and/or peripheral subunit-binding domains or lipoyl domains (arrows) attached to the E1p and/or E3.

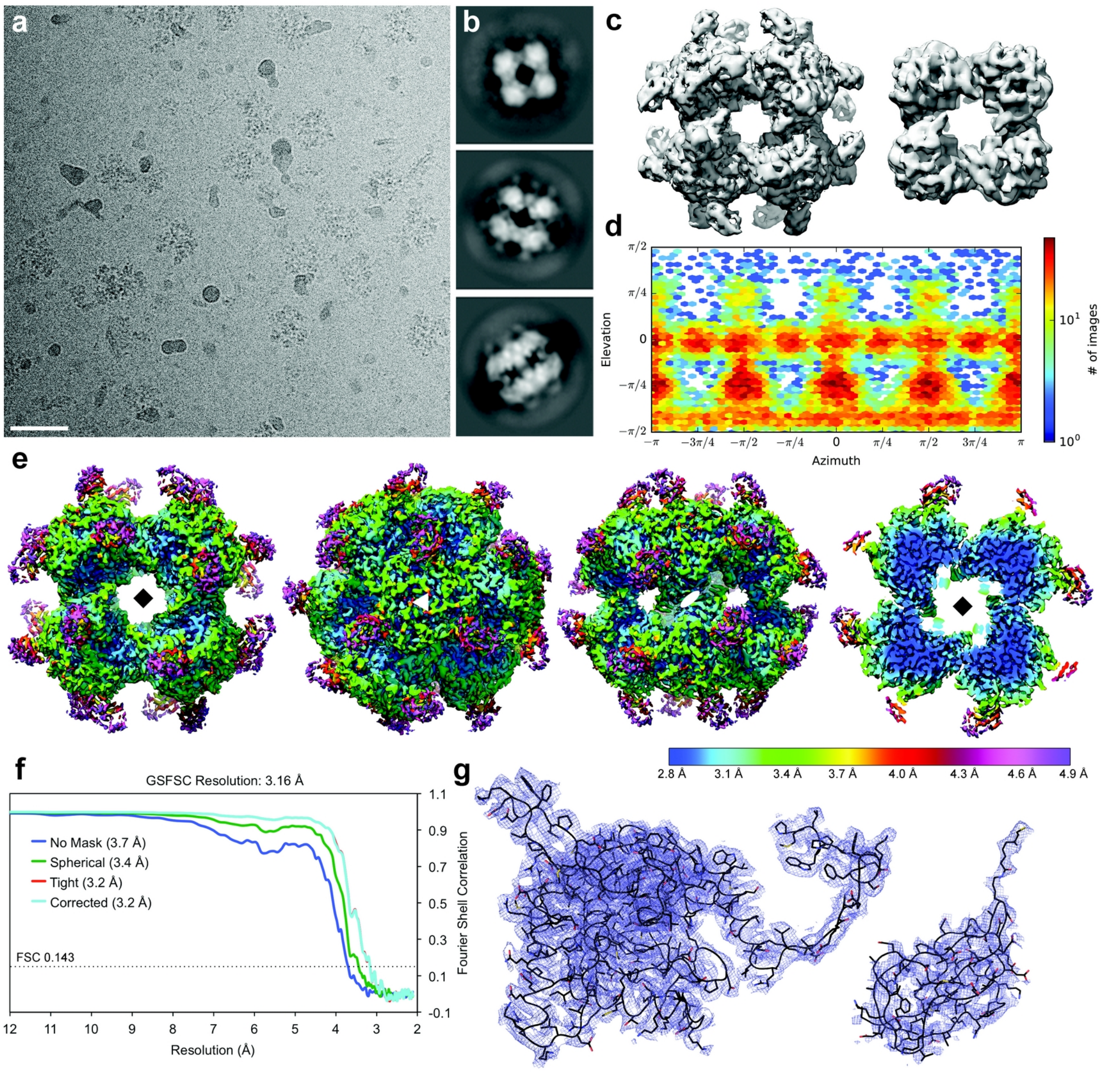

Supplementary Fig. 5 Cryo-EM reconstruction of the *E. coli* pyruvate dehydrogenase complex E2p core. a A representative micrograph (out of 20,078 micrographs used for particle picking); the scale bar represents 50 nm. **b** 2D classes selected for the initial 3D classification and refinement. **c** 3D classes from heterogeneous 3D refinement (3D classification); about 30% of particles were excluded from further refinement as they lacked the lipoyl domains (the class on the right) and 70% of particles were refined further (the class on the left). **d** Angular distribution of particle projections. The heat map shows number of particles for each viewing angle. **e** Final refined and sharpened map for the *E. coli* PDHc E2p core colored according to local resolution estimate (FSC = 0.5) and contoured at 3 σ threshold. Overall surface views along the 4-fold, 3-fold, and 2-fold axes, and a view along the 4-fold axis sliced through the protein interior are show from left to right, respectively. **f** GSFSC plot from the validation of the final refined map. **g** Fit of the final atomic model into the final sharpened cryo-EM map for the E2p catalytic domain monomer (left) and the lipoyl domain (right).

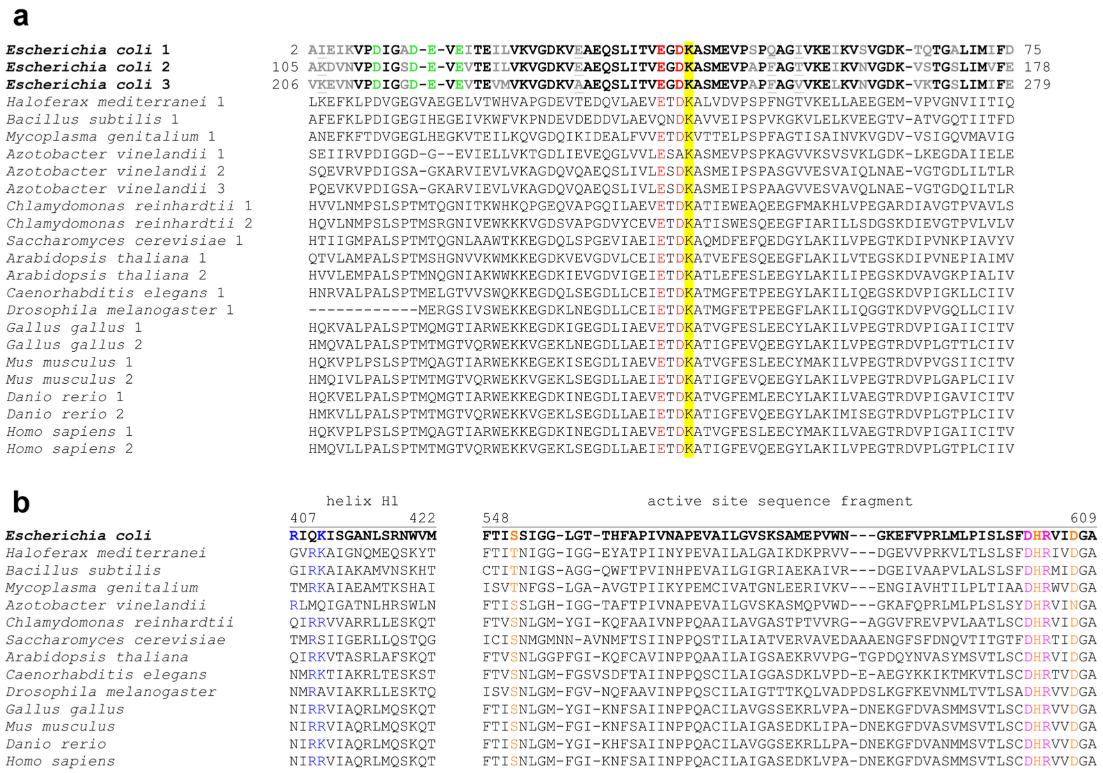

Supplementary Fig. 6 Alignment of selected E2p sequences from all domains of life. a Alignment of the lipoyl domains. Multiple sequences from the same organism correspond to multiple tandem lipoyl domains and are numbered by Arabic numbers from the N-terminus. The patch of acidic residues conserved in the *E. coli* lipoyl domains is highlighted in green, acidic residues conserved in all domains of life are highlighted in red, and the lysine residue carrying the lipoic acid is highlighted in yellow. **b** Alignment of key sequence fragments (helix H1 and the active site fragment) from the catalytic domain. Conserved basic residues at the N-terminus of helix H1 are highlighted in blue, catalytic residues in orange, and the conserved salt-bridge residues that stabilize the catalytic histidine are shown in magenta. Residue numbers corresponding to the *E. coli* sequences are indicated. The sequences were obtained from the Uniprot database (www.uniprot.org).
