# Supplementary material for: Structure of the native pyruvate dehydrogenase complex reveals the mechanism of substrate insertion
Source: Nat Commun. 2021 Sep 6;12:5277. doi: 10.1038/s41467-021-25570-y (PMC8421416; doi:10.1038/s41467-021-25570-y)
Supplement: Supplementary file 3 — Reporting Summary [file 41467_2021_25570_MOESM3_ESM.pdf]

nature research

Corresponding author(s): Pål Stenmark

Last updated by author(s): Aug 4, 2021

Reporting Summary

Nature Research wishes to improve the reproducibility of the work that we publish. This form provides structure for consistency and transparency in reporting. For further information on Nature Research policies, see our Editorial Policies and the Editorial Policy Checklist.

Statistics

For all statistical analyses, confirm that the following items are present in the figure legend, table legend, main text, or Methods section.

n/a	Confirmed	
☐	☒	The exact sample size (n) for each experimental group/condition, given as a discrete number and unit of measurement
☒	☐	A statement on whether measurements were taken from distinct samples or whether the same sample was measured repeatedly
☒	☐	The statistical test(s) used AND whether they are one- or two-sided<br>*Only common tests should be described solely by name; describe more complex techniques in the Methods section.*
☒	☐	A description of all covariates tested
☒	☐	A description of any assumptions or corrections, such as tests of normality and adjustment for multiple comparisons
☒	☐	A full description of the statistical parameters including central tendency (e.g. means) or other basic estimates (e.g. regression coefficient) AND variation (e.g. standard deviation) or associated estimates of uncertainty (e.g. confidence intervals)
☒	☐	For null hypothesis testing, the test statistic (e.g. F, t, r) with confidence intervals, effect sizes, degrees of freedom and P value noted<br>*Give P values as exact values whenever suitable.*
☒	☐	For Bayesian analysis, information on the choice of priors and Markov chain Monte Carlo settings
☒	☐	For hierarchical and complex designs, identification of the appropriate level for tests and full reporting of outcomes
☒	☐	Estimates of effect sizes (e.g. Cohen's d, Pearson's r), indicating how they were calculated

Our web collection on statistics for biologists contains articles on many of the points above.

Software and code

Policy information about availability of computer code

Data collection	EPU, version 2.7
Data analysis	cryoSPARC 2.15, Coot 0.8.9.1, Phenix 1.16, CCP-EM 1.4.1, UCSF Chimera version 1.13.1, PyMOL Molecular Graphics System version 2.2.3

For manuscripts utilizing custom algorithms or software that are central to the research but not yet described in published literature, software must be made available to editors and reviewers. We strongly encourage code deposition in a community repository (e.g. GitHub). See the Nature Research guidelines for submitting code & software for further information.

Data

Policy information about availability of data

All manuscripts must include a data availability statement. This statement should provide the following information, where applicable:

- Accession codes, unique identifiers, or web links for publicly available datasets
- A list of figures that have associated raw data
- A description of any restrictions on data availability

Source data are provided with this paper. The cryo-EM reconstruction has been deposited in the Electron Microscopy Data Bank under the EMDB accession code EMD-12104 (https://www.ebi.ac.uk/emdb/entry/EMD-12104) and the atomic model has been deposited in the Protein Data Bank under the PDB accession code 7b9k (https://doi.org/10.2210/pdb7B9K/pdb).

Field-specific reporting

Please select the one below that is the best fit for your research. If you are not sure, read the appropriate sections before making your selection.

☒ Life sciences ☐ Behavioural & social sciences ☐ Ecological, evolutionary & environmental sciences

For a reference copy of the document with all sections, see nature.com/documents/nr-reporting-summary-flat.pdf

Life sciences study design

All studies must disclose on these points even when the disclosure is negative.

Sample size	A total of 20,133 cryo-EM movies were recorded. The cryo-EM data collection was planned for a total of 4 days of data acquisition, which should, from our general experience, yield enough data for a reconstruction of the structure of the complex. A successful determination of the structure at high resolution proved that the amount of collected data was sufficient.
Data exclusions	All movies were analyzed and 55 movies were excluded from further analysis due to low quality and ice artifacts.
Replication	All replication attaempts (protein preparation, cryo-EM grid preparation, data collection and analysis) were successful. A total of 5 purifications were performed, a total of 20 cryo-EM grids were prepared in three independent sessions, the cryo-EM data were collected in a total of 3 independent data collection sessions, and the data processing was performed iteratively, analyzing each data collection batch separately and combining all collected data together in the analysis.
Randomization	No randomization was relevant for this study. Randomization is not relevant for this type of structural biology work. The collected cryo-EM data are random by nature.
Blinding	Blinding is not relevant for this type of structural study. A big part of the data processing is based on computer automation and only one type of input data is used (protein complex particles), so there is no risk of bias induced by a lack of blinding.

Reporting for specific materials, systems and methods

We require information from authors about some types of materials, experimental systems and methods used in many studies. Here, indicate whether each material, system or method listed is relevant to your study. If you are not sure if a list item applies to your research, read the appropriate section before selecting a response.

Materials & experimental systems

n/a	Involved in the study
☐	☒ Antibodies
☒	☐ Eukaryotic cell lines
☒	☐ Palaeontology and archaeology
☒	☐ Animals and other organisms
☒	☐ Human research participants
☒	☐ Clinical data
☒	☐ Dual use research of concern

Methods

n/a	Involved in the study
☒	☐ ChIP-seq
☒	☐ Flow cytometry
☒	☐ MRI-based neuroimaging

Antibodies

Antibodies used	The primary anti-lipoate antibody was a gift from Dr. Luke Szweda.<br><br>The secondary antibody was horseradish peroxidase-conjugated goat anti-rabbit antibody, cat no. #1705046, Bio-Rad, Hercules, CA, USA
Validation	The primary anti-lipoate antibody was a gift from Dr. Luke Szweda and the work with this antibody in his laboratory can be considered the validation of the antibody (Humphries and Szweda, 1998, Biochemistry 37, 15835-15841).<br><br>The secondary antibody was horseradish peroxidase-conjugated goat anti-rabbit antibody, cat no. #1705046, Bio-Rad, Hercules, CA, USA
